# New transcription regulatory mechanisms of latent HIV LTR

**DOI:** 10.1186/1742-4690-9-S1-O3

**Published:** 2012-05-25

**Authors:** Haleh Rafati, Yuri Moshkin, Tokameh Mahmoudi, Maribel Parra, Shweta Hakre, Eric Verdin

**Affiliations:** 1 Department of Biochemistry, Erasmus University Medical Centre, Rotterdam, The Netherlands; 2 Cancer Epigenetics and Biology Program (PEBC), IDIBELL, Barcelona, Spain; 3 Gladstone Institute of Virology and Immunology, UCSF, San Francisco, CA, USA

## 

Despite the effectiveness of antiretroviral medication, the HIV virus persists in resting memory T cells of infected patients in a latent state, providing the main impediment to eradication of the virus. We are interested in identifying the molecular mechanism responsible for the establishment and maintenance of HIV latency and its re-activation. We recently used a cell system reflecting HIV latency in my lab to determine the high resolution nucleosomal landscape of the latent HIV LTR and examine its dynamic changes upon re-activation (Rafati et al., Nov 2011 PLoS Biology). We combined mathematical predictions of nucleosome positioning with a combinatorial biochemical approach based on formaldehyde crosslinking of latent and activated HIV infected cells (using FAIRE, ChIP and high resolution MNase nucleosomal mapping) to define LTR nucleosome positioning and regulation during active and latent HIV infections. We found that BAF, an ATP-dependent chromatin remodelling complex generates a chromatin structure at the LTR that is energetically unfavorable to its intrinsic histone-DNA sequence preferences. Specifically, we find that BAF positions a repressive nucleosome immediately downstream of the HIV transcription start site, abrogating transcription, and in this way contributes to the establishment and maintenance of HIV latency. Our data describe a novel molecular mechanism for the establishment and maintenance of HIV latency, and we identify the catalytic subunit of BAF, the enzyme BRG1, as a putative molecular target to deplete the latent reservoir in infected patients. We will also present preliminary data addressing the role of a novel signalling pathway in de-repression of latent HIV, and the effect of small molecules and ligands, which activate this pathway to study reactivation of latent HIV. We anticipate these experiments will further our understanding of HIV transcription regulation and identify both novel cofactors for targeting and molecules with potential to purge HIV latency.

